# Large Language Models as a Rapid and Objective Tool for Pathology Report Data Extraction

**DOI:** 10.5146/tjpath.2024.13256

**Published:** 2024-05-18

**Authors:** Beyza Bolat, Ozgur Can Eren, A. Humeyra Dur Karasayar, Cisel Aydın Mericoz, Cigdem Gunduz Demir, Ibrahim Kulac

**Affiliations:** Koc University School of Medicine, Koc University, Istanbul, Turkey; Department of Pathology, School of Medicine, Koc University, Istanbul, Turkey; Graduate School of Health Sciences, Koc University, Istanbul, Turkey; Koc University IsBank Research Center for Infectious Diseases, Istanbul, Turkey; Graduate School of Health Sciences, Koc University, Istanbul, Turkey; Koc University & Is Bank Artificial Intelligence Center, Istanbul, Turkey; Department of Pathology, School of Medicine, Koc University, Istanbul, Turkey; Koc University School of Medicine, Koc University, Istanbul, Turkey; Koc University & Is Bank Artificial Intelligence Center, Istanbul, Turkey; Department of Computer Engineering, Koc University, Istanbul, Turkey; Department of Pathology, School of Medicine, Koc University, Istanbul, Turkey; Graduate School of Health Sciences, Koc University, Istanbul, Turkey; Research Center for Translational Medicine, Koc University, Istanbul, Turkey

**Keywords:** Large language models (LLMs), Pathology, Generative pre-trained transformer-4 (GPT-4), ChatGPT, Bard

## Abstract

Medical institutions continuously create a substantial amount of data that is used for scientific research. One of the departments with a great amount of archived data is the pathology department. Pathology archives hold the potential to create a case series of valuable rare entities or large cohorts of common entities. The major problem in creation of these databases is data extraction which is still commonly done manually and is highly laborious and error prone. For these reasons, we offer using large language models to overcome these challenges. Ten pathology reports of selected resection specimens were retrieved from electronic archives of Koç University Hospital for the initial set. These reports were de-identified and uploaded to ChatGPT and Google Bard. Both algorithms were asked to turn the reports in a synoptic report format that is easy to export to a data editor such as Microsoft Excel or Google Sheets. Both programs created tables with Google Bard facilitating the creation of a spreadsheet from the data automatically. In conclusion, we propose the use of AI-assisted data extraction for academic research purposes, as it may enhance efficiency and precision compared to manual data entry.

**Dear Editor,**

Medical institutions provide indispensable resources for both patient care and academic research. Pathology archives in this regard offer the opportunity to study common entities (prostate cancer, colorectal cancer, invasive breast cancer, etc.) or to create case series of valuable rare entities. Although resources are available, scanning through each case on electronic databases and extracting data from the selected ones are time-consuming and constitute a major bottleneck in the creation of datasets. Manually reviewing and extracting data from electronic databases is not only time-consuming but also very error prone as most data extraction is being done by copying and pasting or reading and writing small pieces of data one by one. We therefore offer an AI-based method for semi-automatic data extraction for pathologists who run pathology data driven research projects.

Large language models (LLMs) are complex algorithms with the ability to process and generate written or spoken language. After the introduction of GPT3.5 to the public, LLMs became extremely popular and the entire population including researchers in many fields also began to explore its capabilities. LLMs have rapidly established their prominence in numerous sectors, including banking and marketing, by significantly reducing the burden of labor-intensive tasks. One of the essential skills of LLMs is their ability to convert texts into structured formats, which can be used for any type of documentation. There is increasing interest in LLMs and their potential use in clinical practice as well as educational and research purposes (1–3). In this regard, the use of LLMs can be a game changer in handling the laborious nature of pathologic data-oriented database creation in medical institutions with a high volume of annual biopsies. They not only save time but also create standardized documents, a task that is challenging when done manually. By using LLMs to extrapolate data from pathology reports, we aim to automate data extraction for research, overcoming the impediments of manual dataset creation and minimizing human errors. Medical researchers worked on the implementation of different transformer architectures (BioBERT, BioMegatron, etc.) for complex tasks such as natural language inference, medical question answering or clinical concept extraction and had promising results (4). Distinct from past research and proposals, we employed publicly available and easy-to-use chatbot-based LLMs, namely ChatGPT (OpenAI Inc, San Francisco, CA, USA) and Google Bard (Alphabet Inc, Googleplex, Mountain View, CA, USA), to assess their efficiency on surgical pathology data extraction.

Pathology reports of selected resection specimens were retrieved from the electronic archives of Koç University Hospital. Ten pathology reports of radical prostatectomy specimens were selected for the initial set. These reports were signed out by three different pathologists. Despite minor variations, all reports were following a similar format, respecting almost all the fields CAP recommends in a prostatectomy pathology report. After de-identification of the cases, the reports were simply transferred (copy and paste) to ChatGPT with the following command:

“*Here are pathology reports of prostate cancer patients. I need them in a synoptic report format as an excel spreadsheet to store uniform data of these patients. Although the reports are in Turkish, I need every output in English. Every patient’s report starts with B##-#####*.”

The outputs were downloaded and verified by two pathologists independently ensuring the data were correct.

The output of ChatGPT had a table-like structure but required copy-and-pasting into a spreadsheet. Hence, we created the following table in Microsoft Excel (Table 1). We ran the same command with Google’s Bard and it automatically created a spreadsheet upon receiving the appropriate command. Presented below is the output produced by Bard using a similar prompt to ChatGPT’s. This spreadsheet can also be exported to Google Sheets (Table 2) using a built-in export function.

**Table 1:** Extracted data from radical prostatectomy specimens using OpenAI’s ChatGPT

**Patient ID**	**Surgery Type**	**Prostate Tumor Location**	**Gleason Score**	**Tumor Volume**	**Extra-capsular Extension**	**Seminal Vesicle Invasion**	**Surgical Margins**	**Lymph Node Involvement**	**Pathological Stage**
**Case 1**	Radical Prostatectomy	Left posterior lateral	6 (3+3)	1%	No	No	Intact	No	pT2N0 (R0)
**Case 2**	Radical Prostatectomy	Left lobe	7 (3+4)	25-30%	No	No	Intact	No	pT2 (R0)
**Case 3**	Radical Prostatectomy	Right posterior	7 (4+3)	15%	Yes	No	Intact	No	pT3a (R0)
**Case 4**	Radical Prostatectomy	Bilateral anterior	7 (3+4)	5%	No	No	Positive	No	pT2(+) (R1)
**Case 5**	Radical Prostatectomy	Right anterior	7 (3+4)	<5%	No	No	Negative	No	pT2N_M_
**Case 6**	Radical Prostatectomy	Right lateral	7 (3+4)	<5%	No	No	Negative	No	pT2N_M_
**Case 7**	Radical Prostatectomy	Left posterolateral	9 (5+4)	5%	Yes	Yes	Negative	No	pT3b (R0)
**Case 8**	Radical Prostatectomy	Right anterolateral	6 (3+3)	<5%	No	No	Negative	No	pT2N_M_
**Case 9**	Radical Prostatectomy	Bilateral	8 (5+3)	50%	Yes	Yes	Positive	No	pT3b (R1)
**Case 10**	Radical Prostatectomy	Left posterior lateral	7 (4+3)	5%	Yes	No	Positive	No	pT3aN0 (R1)

Parts of the extracted data are incomplete because the original report did not contain all the necessary information

**Table 2:** Extracted data from radical prostatectomy specimens using Google Bard

**Variable**	**Patient 1**	**Patient 2**
**Specimen ID**	Case 1	Case 2
**Operation type**	Robotic radical prostatectomy + bilateral pelvic lymphadenectomy	Robotic radical prostatectomy
**Prostate**	Multifocal - bilateral	Unifocal - left lobe
**Dominant tumor location**	Left lobe posterolateral	Left lobe, both anterior and posterior quadrants
**Dominant tumor volume**	1%	25-30%
**Dominant tumor subtype**	Acinar	Acinar
**Gleason score**	6 (3+3)	7 (3+4)
**Intraductal carcinoma**	Not seen	Not seen
**Extraprostatic extension**	Not seen	Not seen
**Lymphovascular invasion**	Not seen	Not seen
**Seminal vesicles**	Intact	Intact
**Surgical margins**	Intact	Intact
**Pathological stage**	pT2N0 (R0)	pT2 (R0)

Parts of the extracted data are incomplete because the original report did not contain all the necessary information

A similar procedure was carried out for other specimen types (colectomy specimens and pancreatectomy specimens) to assess the models’ applicability (results not shown).

It is important to highlight our iterative approach in handling multiple reports. Due to the limitations, reports were presented individually or in small groups. Although a standardized data table can be readily generated using Bard, this was not the case for ChatGPT. Bard also facilitates the creation of spreadsheets without necessitating manual intervention. However, it is worth noting that Bard encountered challenges processing data from 10 cases, so the practice that we present here was limited to two cases. It is worth noting that while integrating a software into the laboratory information system could yield smoother data extraction, most pathologists probably do not have the expertise to undertake such technical operations.

Generating datasets for academic use from pathology reports can be complex, particularly when researchers deal with diverse data sources of varying formats. GPT-based LLMs offer a potential solution for extracting data objectively and uniformly from reports, usually free of charge. These AI tools can understand complex medical terminology and convert data into desired formats (mostly), irrespective of the input and output languages. Although many institutions try to update their reporting systems into a modern synoptic format that is built in the laboratory information system (LIS), archival data would still need manual attention. In our institute, reaching uniformity in pathology reports is practically impossible due to software limitations and individual reporting preferences.

In conclusion, the versatility of this technology extends beyond pathology records to encompass various forms of medical data, as long as they undergo de-identification and anonymization for the creation of structured datasets. We advocate for the transition from manual data entry to an AI-assisted rapid data collection approach, particularly for academic research. This method promises enhanced efficiency and precision. The burgeoning integration of AI in medicine, as evidenced by our work, is a harbinger of a transformative era. In the near future, we anticipate a substantial proliferation of AI-assisted applications across multiple domains of medical science, signaling a paradigm shift in healthcare innovation and delivery.

## Conflict of Interest

All the authors declare that they have no competing interests.
